# Innovative Therapies against Human Glioblastoma Multiforme

**DOI:** 10.5402/2011/787490

**Published:** 2011-07-24

**Authors:** Annamaria Cimini, Rodolfo Ippoliti

**Affiliations:** Department of Basic and Applied Biology, University of l'Aquila, Via Vetoio No. 10, 67010 L'Aquila, Italy

## Abstract

Glioblastoma multiforme is the most invasive and aggressive brain tumor in humans, and despite the latest chemical and radiative therapeutic approaches, it is still scarcely sensitive to these treatments and is generally considered an incurable disease. This paper will focus on the latest approaches to the treatment of this cancer, including the new chemicals such as proautophagic drugs and kinases inhibitors, and differentiating agents. In this field, there have been opening new perspectives as the discovery of possible specific targets such as the EGFRvIII, a truncated form of the EGF receptor. Antibodies against these targets can be used as proapoptotic agents and as possible carriers for chemicals, drugs, radioisotopes, and toxins. In this paper, we review the possible mechanism of action of these therapies, with particular attention to the combined use of toxic substances (for example, immunotoxins) and antiproliferative/differentiating compounds (i.e., ATRA, PPAR*γ* agonists). All these aspects will be discussed in the view of progress clinical trials and of possible new approaches for directed drug formulations.

## 1. Cellular and Molecular Biology of Gliomas

Malignant gliomas, the most common type of primary brain tumor, are a spectrum of tumors of varying differentiation and malignancy grades. Early genetic events differ between astrocytic and oligodendroglial tumors, but all tumors have an initially invasive phenotype that does not allow simple therapeutic approaches. Progression-associated genetic alterations are common to different tumor types and target growth-promoting and cell-cycle-controlling pathways, resulting in focal hypoxia, necrosis, and angiogenesis. Mutations in the retinoblastoma protein (Rb) have been identified in 20% of malignant gliomas [1] and those lacking mutations in Rb contain mutations in other molecules involved in the Rb signaling pathway, such as the cell-cycle regulator p16INK4A or cyclin-dependent kinase (CDK). 60%–80% of anaplastic astrocytoma contains homozygous deletion, mutation, and promoter hypermethylation of the INK4A/ARF locus, and 25% of anaplastic oligodendrogliomas have hypermethylation of the INK4A/ARF locus [2]. In addition, gene amplification in gliomas causes the overexpression of several mitogens and their specific receptors. These include epidermal growth factor (EGF), platelet-derived growth factor (PDGF), insulin-like growth factor 1 (IGF-1), and their specific receptors (EGFR, PDGFR, and IGFR), all of which are involved in autocrine or paracrine signaling in gliomas [3–7]. These receptors with tyrosine kinase activity also exist in constitutively active mutant forms in gliomas [7], regulating several signaling pathways such as phosphoinositide-3-kinase/AKT-protein kinase B (PI3K/AKT-PKB), RAS/mitogen-activated protein kinase (MAPK), and phospholipase C/protein kinase C (PLC/PKC). These signaling pathways control several biological processes, such as cell proliferation, differentiation, invasion, and apoptosis [8]. Phosphatase/tensin homolog protein (PTEN), which acts as a tumor suppressor by inhibiting the PI3K/AKT signaling pathway, can also be involved in gliomagenesis through loss-of-function mutations [9, 10]. In gliomas, several overexpressed angiogenic factors, such as fibroblast growth factor (FGF), interleukin (IL)-8, PDGF, transforming growth factor (TGF), and vascular endothelial growth factor (VEGF), have been identified. Combined genetic alterations in these factors result in aggressive cellular proliferation, invasion, and angiogenesis rendering malignant gliomas resistant to intensive therapy. Recently, a population of glioma stem cells has been isolated. This subpopulation of stem-like cells plays an important role in the tumorigenic process [11–14]. Because glioma stem cells can self-propagate, it might also be important to specifically target glioma stem cells to avoid recurrence of the glioma [15]. The possibility to isolate GBM stem cells opens the frontier of gene replacement, knockdown, or silencing as a new therapeutic approach [15].

## 2. Chemotherapy

In standard treatment protocols, brain tumor resection and radiation therapy are followed by chemotherapy with drugs causing DNA alkylation, like nitrosoureas. Standard treatment is a combination of procarbazine, lomustine and vincristine or carmustine or temozolomide alone [16]. Recently, GLIADEL wafers have been introduced. GLIADEL wafers are small, dime-sized biodegradable polymer wafers that are designed to deliver BCNU or carmustine directly into the surgical cavity created when a brain tumor is resected. Immediately after a neurosurgeon operates to remove the high-grade malignant glioma, up to eight wafers are implanted along the walls and floor of the cavity that the tumor once occupied. Each wafer contains a precise amount of carmustine that dissolves slowly, delivering carmustine to the surrounding cells. A clinical study was conducted in 240 men and women undergoing surgery for a newly diagnosed high-grade malignant (cancerous) glioma [17]. Each patient was randomly assigned to receive either surgery with implantation of GLIADEL followed by radiation therapy, or surgery with implantation of placebo wafers (wafers without any carmustine) followed by radiation therapy. The results of this study showed that survival was prolonged in the patients who received GLIADEL wafers compared to those who received the placebo wafers; median survival increased to 13.8 months from 11.6 months. Until recently, the benefit of chemotherapy following surgery and radiation has been almost negligible for most GBM patients. Autophagy represents an alternative tumor-suppressing mechanism to overcome, at least partly, the dramatic resistance of many cancers to radiotherapy and proapoptotic-related chemotherapy. Temozolomide contributes significant therapeutic benefits in glioblastoma patients [18]. Indeed, the addition of temozolomide to radiotherapy resulted in a longer median survival time in newly diagnosed glioblastoma patients, 14.6 versus 12.1 months, and a higher 2-year survival rate, 26.5% versus 10.4% [19]. Part of temozolomide cytotoxic activity is exerted through proautophagic processes, at least in glioblastoma cells, as a result of the formation of O6-methylguanine in DNA, which mispairs with thymine during the following cycle of DNA replication [16]. Glioma cells, thus, respond to temozolomide by undergoing G2/M arrest, but ultimately die from autophagy [19, 20]. Knowing that O6-alkylguanine-DNA alkyltransferase (AGT) is a DNA repair enzyme that limits the efficacy of temozolomide in glioblastoma cells [20] first showed that inhibition of AGT by O6-benzylguanine can render previously resistant glioblastoma cells sensitive to temozolomide. The data obtained by Hegi et al. [20] show that GBM patients with a methylated O6-methylguanine-DNA methyltransferase (MGMT) promoter benefited from temozolomide, while those who did not were less responsive. This case was recently followed up by a randomized, Phase III study including 573 GBM patients. Patients treated with temozolomide after radiation had a median survival of 14.6 months as compared to 12.1 months for patients given radiotherapy alone [20]. These results made this treatment scheme to become the standard of care for patients with GBM and approved in USA and Europe for newly diagnosed GBM. Almost all treated high-grade astrocytoma cases recur and the tumor usually arises within 2 cm of the prior resection margin [21]. The current treatment strategies for recurrent astrocytoma have recently been reviewed [22]. Available therapies following progression are considered ineffective with a progression free survival after six months (PFS-6) of less than 15%. That is now the commonly used end point to assess therapeutic activity in clinical oncology of recurrent glioblastomas [23, 24].

## 3. Natural Resistance of Glioblastoma Cells to Apoptosis

Resistance to apoptosis is considered to be a characteristic of many diverse cancer cells [25]. Defects in apoptosis underlie not only tumorigenesis but also resistance to cancer treatments [25]. Furthermore, the inherent resistance of cancer cells to radiotherapy and chemotherapy is mainly due to changes at genomic, transcriptional, and posttranscriptional levels of proteins and protein kinases and their transcriptional factor effectors [25]. The phosphatase and tensin homologue deleted on chromosome ten (PTEN)/phosphatidylinositol 3P-kinase (PI3K)/Akt/mammalian target of rapamycin (mTOR)/nuclear factor kappa B (NF-*κ*B) and the Ras/Raf/mitogen-activated protein kinase extracellular signal-related kinase (ERK) kinase (MEK)/ERK signaling cascades play critical roles in the regulation of gene expression and in the prevention of apoptosis [26]. Components of these pathways are mutated or aberrantly expressed in human cancer (e.g., Ras, B-Raf, PI3K, PTEN, and Akt) [26]. An aberrantly activated PI3K/Akt pathway renders tumor cells resistant to cytotoxic insults, including those related to proapoptotic anticancer drugs [26–32]. Deregulation of the PTEN/PI3K/Akt pathway has been associated with resistance to chemotherapeutic drugs used in breast cancer, prostate cancer, ovarian cancer, and malignant gliomas therapy [26–31]. Shingu et al. [33] have shown, in the context of glioma cells, that the inhibition of this pathway restores or even augments the effectiveness of chemotherapy. Preclinical studies suggested that sensitivity to mTOR inhibitors may correlate with activation of the PI3K pathway and/or with aberrant expression of cell-cycle regulatory or antiapoptotic proteins. mTOR inhibitors are currently under evaluation in clinical trials and include rapamycin (sirolimus) and the related derivatives temsirolimus (CCI-779), everolimus (RAD001), and AP23573 [34]. These trials have shown that mTOR inhibitors are well tolerated and may induce prolonged stable disease and tumor regressions in cancer patients [34]. Apoptosis-based therapies gained interest as promising experimental treatment strategies, since direct induction of apoptotic cell death can overcome many of the classical resistance mechanisms such as activated DNA repair or detoxification. The death ligand TRAIL/Apo2L might be a useful tool to trigger apoptosis in cancer, since TRAIL kills tumor cells of diverse cellular origin without severe toxic side effects [35, 36]. However, despite the common expression of death receptors, not all glioblastoma cells are susceptible to TRAIL due to intracellular blockage of apoptotic signalling cascades. Therefore, overcoming apoptosis resistance becomes an urgent need in order to sensitize tumors to the actions of death receptor-targeting therapy. A novel group of peroxisomal proliferator activated receptor *γ* (PPAR*γ*) modulating agents sensitize tumor cells to TRAIL-induced apoptosis [37]. One of these drugs, troglitazone, is an oral antidiabetes drug, which belongs to the group of thiazolidinediones. It has been reported that glioblastoma cells are sensitized to TRAIL-induced apoptosis by troglitazone via various mechanisms. Troglitazone led to a marked downregulation of the antiapoptotic proteins FLIP and survivin. Moreover, in some cell lines, the cell surface expression of agonistic and antagonistic TRAIL receptors was altered towards a higher susceptibility to death receptor induced apoptosis. Troglitazone could counteract the capability of tumor cells to become resistant to apoptosis by modulating the apoptotic machinery at different levels [37]. Constitutive activation of the NF-*κ*B pathway also enables cancer cells to resist cytotoxic insults [38]. These changes also affect tumor necrosis factor, Fas, and TRAIL receptors, which play important roles in tumor resistance to apoptosis during cancer progression [36].

## 4. Necrosis

While highly resistant to therapeutic apoptotic stimuli, GBM tumor cells exhibit the paradoxical propensity for extensive cellular necrosis. Necrosis, in fact, is the most prominent form of spontaneous cell death in GBM, presented as foci of necrosis surrounded by broad hypercellular zones contiguous with normal tissue or by parenchymal infiltrates [39]. While limited blood supply and anoxia due to a microthrombotic process has been identified as an important cause of necrosis, the molecular basis for this necrotic phenotype, particularly in the context of intense apoptotic therapy resistance, has recently received attention with the discovery and characterization of the Bcl2-like 12 (Bcl2L12) protein. Bcl2L12 has been shown to be a potent inhibitor of apoptosis that is significantly overexpressed in primary GBMs [40, 41]. Bcl2L12 is a proline-rich protein characterized by a C-terminal 14-amino-acid sequence with significant homology with the BH (Bcl-2 Homology) 2 domain found in several members of the Bcl-2 protein family [37]. RNAi-mediated knockdown of Bcl2L12 sensitizes human glioma cell lines to drug-induced apoptosis and reduces tumor formation in an orthotopic transplant model in vivo [40]. The anti-apoptotic actions of Bcl2L12 is due to its ability to neutralize effector caspase activity likely through specific interaction with effector caspase-7 [42]. These activities of Bcl2L12 are relevant to the necrotic process in the light of studies demonstrating that the suppression of caspase activity redirects the death program from apoptosis to necrosis [42] suggesting that post-mitochondrial caspase activation may act as a molecular switch between apoptosis and necrosis. In support of this findings, germline deletion of post-mitochondrial apoptosis signaling components, such as the caspase activator Apaf-1, or blockage of effector caspase by pan-specific caspase inhibitors results in decreased apoptosis, with concomitant increase of necrosis [42]. On this basis, the upregulation of Bcl2L12 as a novel regulator of the apoptosis/necrosis balance in glial cells may represent an important event in malignant glioma pathogenesis.

## 5. Altered Pathways and Targeted Therapies for Gliomas

### 5.1. Growth Factor Pathways

Platelet-derived growth factor (PDGF) and epidermal growth factor (EGF) are ligands for tyrosine kinase receptors with crucial roles in brain tumor development. Other growth factors involved in brain tumors are insulin-like growth factors, IGFs [43] fibroblast growth factor 2, FGF2 [44], ciliary neurotrophic factor, CNTF [45], hepatocyte growth factor/scatter factor, HGF/SF [46], vascular endothelial growth factor, VEGF [47], and transforming growth factor-*β*, TGF-*β* [48, 49]. The most studied is the epidermal growth factor receptor (EGFR) that was early recognized in gliomas [50, 51]. PDGF is also a mitogen of neural stem cells of embryonic [52] and adult [53] origin. Analysis of expression of PDGF ligands and receptors in human gliomas suggests that there is an autocrine stimulatory loop in almost all gliomas [54].

### 5.2. EGFR Targeting

Concerning the epidermal growth factor receptor (also known as ErbB1 or HER1), is usually over-activated in most human tumors, and particularly in gliomas mostly due to gene amplification [4, 51, 55, 56] leading to a marked enhancement of cellular motility, invasion, and proliferation. It is a transmembrane protein of 170 kDa containing three different domains: one that is responsible of the binding of the growth factor (EGF) on the extracellular side, one that is a transmembrane lipophilic domain, and an intracellular tyrosine kinase domain. The activation of the receptor upon binding of the ligand requires its dimerization causing a molecular structural rearrangement inducing cross-phosphorylation of the two intracellular domains on sites mainly located at the C-terminus of the protein [57, 58]. This process leads to the activation of downstream signaling pathways, which involve several kinases and particularly the effectors mTOR and MAPK. Inhibitors of these enzymes have been produced and tested in several clinical trials. The most studied are inhibitors of the tyrosine kinase activity of EGFR, such as gefitinib [59, 60] and erlotinib [59–65]. Their action is due to competition with ATP in the catalytic site of the tyrosine kinase domain, and hence, the inhibition of the downstream phosphorylation activities. Their degree of success was relatively low and heterogeneous if compared with the same treatment in other cancer forms, that is, lung cancer [66]. A very promising and interesting evolution of the EGFR targeting is due to the discovery that many forms of glioblastoma express several splicing variants of the receptor, among which the most represented is that called EGFRvIII lacking a portion of the extracellular domain corresponding to exons 2–7, giving rise to a protein of 145 kDa [67, 68]. This receptor variant is constitutively dimerized, and hence auto-phosphorylated and active, and not able to bind the extracellular ligands. Other forms of mutant receptors for EGF have been described, due to abnormally spliced products or sometime to exon duplications [67, 68]. The spliced products result in the formation of new inter-exon boundaries that represent unique sites differentiating these receptor forms from the wt receptor. In particular EGFRvIII shows a glycine residue at the joining point between exons 1 and 8 that is not present in the wt EGFR. So, the EGFR variants are one of the few examples of glioma-specific targets available for directed therapies. The expression of the variant form vIII seems to be correlated with an increase of tumorigenicity [69], as demonstrated by the fact that transformants obtained by introducing copies of the EGFRvIII in U87MG human and N6 mouse cells allow these cells to develop and proliferate in nude mice, with a consistent decrease of apoptosis [70, 71]. The increased tumorigenic activity of the mutant EGFRvIII has been associated with an increase of activation of Ras-GTP [71, 72] and PI 3-kinase [73] as well as the cJun terminal kinase (JNK), [74], while the reduction of apoptosis has been linked to an upregulation of BclXL [75]. Inhibitors of PI 3-kinase such as wortmannin and LY294002 [73] decreased the transforming activity of EGFRvIII positive cell lines and decreased the activation of JNK, thus suggesting that EGFRvIII acts primarily through the activation of PI 3-kinase/JNK. Therefore, these pathways are expected to be a possible therapeutic target for specific inhibitors. Since EGFRvIII is tumor specific, some groups have developed antibodies raised against the extracellular domain, and particularly to the junction between exons 1–8 that is not present in the wt receptor [76, 77]. Monoclonal antibodies with a good affinity [78] and single chain fragments [78, 79] have been described; the binding of most of these antibodies to EGFRvIII positive cells rapidly induces the receptor internalization, thus offering a potential tool to introduce drugs into cancer cells. Recombinant single chain antibodies (MR1), from a murine phage display library [80], were isolated by selection against a synthetic peptide containing the joining aminoacidic sequence at the boundary of exons 1–8 spliced product. This single chain [80, 81] was engineered and linked to pseudomonas exotoxin A, producing an immunotoxin that is greatly active against glioblastoma cells. After affinity maturation, the scFv-based immunotoxin was more efficient against glioblastoma cells [82]. Recently, [81] it has been demonstrated that an MR1-based immunotoxin containing PE38-KDEL (a truncated version of pseudomonas exotoxin) is able to induce antitumor immunity against the mutated EGFRvIII, notwithstanding its cytotoxic activity against EGFRvIII-positive cells, also in EGFRvIII-negative cells, and that this immunization process is dependent on the presence of CD8+ and CD4+ T cells [83]. Furthermore, antibodies raised against EGFRvIII have been used to target glioblastoma cells, as in the case of the MoAb L8A4 [80] or the scFv MR1-1 [77] radiolabeled with 125I. As EGFRvIII-directed antibodies rapidly induce the internalization of the receptor-antibody complex, some modifications have been suggested to allow retention of the radiolabeled antibody inside and the cell, using polycationic peptides added to the aminoacidic sequence of these molecules capable of trapping them inside lysosomes. The increase of cellular retention may be helpful for the action of the radioisotope in intoxicating the cells [84]. Very recently, an antibody against EGFRvIII was used to deliver iron oxide nanoparticles against model U87 glioblastoma cells [85]. These derivatized nanoparticles were used for MRI contrast enhancement and CED (convection-enhanced delivery) therapy, and significantly reduced the size of intracranial human xenograft tumors and prolonged the life of treated animals. Finally, it should be cited as a new therapy approach the possibility to induce immune responses against the EGFRvIII (reviewed by [85, 86]). This approach started by using immunogenic peptides corresponding to a 14 mer including the boundary sequence between exons 1 and 8 [86] injected into mice as a complex with adjuvants, or the recombinant complete EGFRvIII expressed on the surface of mammalian (300.19 mouse cell line, [87]) cells. In both cases, animals were protected from the insertion and spreading of the injected tumor cells expressing EGFRvIII, being the protective effect due to the presence of CD8+ and CD4+ T cells. Peptides from EGFRvIII could be also used to induce dendritic cell- (DC-) mediated immunization [87, 88] when these cells were treated with an extract of EGFRvIII-transfected cells. Since it has been shown that is possible to isolate DC from the peripheral blood of glioma patients, this could open a concrete possibility to apply combined therapies including DC vaccination for the treatment of glioblastoma in man. Furthermore, recently, a fusion protein EGFRvIII-HBcAg has been used to achieve immune response in BALB/c mice, with a significant increase of the production of INF-*γ*-secreting lymphocytes [89] and remarkable resistance to tumor implantation.

### 5.3. PDGF Targeting

Platelet-derived growth factors are four different polypeptide chains (A to D) that are recognized by two types of receptor (PDGFR*α* and *β*). The binding of PDGF to its receptor induces an activation process very similar to that described for EGFR, with receptor dimerization, autophosphorylation, and transduction of signals through the Ras-MAPK, Pi 3Kinase, Src, Stat, and phospholipase C. The overexpression of PDGF and PDGFR is common in secondary glioblastomas [90] apparently due to the decrease of expression of the tumor suppressor TP53. At the moment, this receptor has been scarcely targeted and mainly by the use of tyrosine kinase inhibitors, such as imatinib, that are not specific to PDGFR and possibly act differently to what observed for EGFR inhibitors, by binding in the proximity of the catalytic site, thus blocking the interaction of protein kinases with their substrates. This inhibitor blocks the activity of several kinases including bcr-abl and c-kit and has been approved for chronic myeloid leukaemia and gastrointestinal tumors, but it proved scarcely effective against gliomas in a phase II trial against grade III gliomas that should be highly dependent on PDGFR [91]. This failure has possibly been attributed to a scarce penetration of the drugs inside the tumor mass or to a reduced role of PDGF in such advanced tumors.

### 5.4. VEGFR Targeting

Vascular endothelial growth factor A is a member of VEGF family and acts with its receptor in tumor angiogenesis, a very complex phenomenon that passes through several steps including the disrupture of the basal membrane, the increase in proliferation of endothelial cells, the interaction between cells and the matrix, and the mobilization of endothelial cells and hematopoietic progenitors [92]. The action of VEGF also induces the expression of other proangiogenic factors such as the urokinase-type plasminogen activator and its overexpressed receptor and metalloproteinase-1. VEGFRs involved in the cellular response to VEGF-A are the forms 1 and 2 (Flt-1 and Flt-2/KDR). VEGFR-2 is a tyrosine kinase inducing signalling via Ras/Raf/MEK/MAPK and PI3K/AKT/PKB and proteine kinase C-pathways. Also, for this receptor, some kinase inhibitors have been tested such as vatalanib (PTK787/ZK222584), sorafenib (BAY 43-9006), sutinib (SU11248), and cediranib (AZD2171). All these inhibitors, although active on glioblastoma cells, proved again relatively efficient during test on phase II trials [93, 94]. More promising is the targeting of VEGFR with a specific humanized monoclonal antibody (bevacizumab) in combination with irinotecan [95–97] that gave a high response into patients with a consistent reduction of the tumor mass although the overall survival was not greatly enhanced, suggesting that blocking cell proliferation and tumor mass reduction are not sufficient to counteract the disease. Further, very recently a novel therapeutic approach has been proposed, targeting the VEGFR expression via the introduction of an artificial transcriptional regulator (Zinc finger, [98]) eventually mediated by an adenovirus [93], inducing a downregulation of VEGFR by acting on the VEGFR promoter, resulting in marked antitumor effect on a human glioblastoma xenograft model. Finally, it should be cited among the unconventional therapies the possibility to target VEGFR by a fusion protein, VEGF121-rGel [99], containing the ribosome inactivating protein gelonin as a cytotoxic agent.

### 5.5. Other Possible Targets

In the framework of targeted toxicity towards glioblastoma, it is worth citing several immunotoxins that have been made fusing growth factors such as interleukins 4 and 13, urokinase and transferrin with toxins. These approaches are mainly based on the fact that receptors for these substances are not or are very poorly expressed in normal brain tissues. Historically transferrin-based immunotoxins have been produced by fusion with diphtheria toxin mutants (CRM107, [100]) that resulted very effective in reducing tumor mass in the brain after direct infusion of the toxin. The high response to the immunotoxin, as in the case of other chimeras containing toxins, is due to the rapid and efficient internalization of the receptors allowing the toxic molecule to be released inside the cell cytoplasm, where the enzyme activity can be obtained (most of the toxins used are protein synthesis inhibitors and thus must reach ribosomes). One of the possible obstacles in using transferrin-based immunotoxin could be the competition of free transferrin in the serum that could be overcome by the use of monoclonal antibodies raised against the transferrin receptor that do not recognize epitopes in the ligand-binding domain [100]. Many gliomas and particularly GBM overexpress the IL4 receptor, thus providing a good candidate for the targeting with fusion immunotoxins and indeed very efficient targeting has been reached by intratumoral infusion of a circularly permuted IL4 fused to the catalytic domain of pseudomonas exotoxin (PE38KDEL, [101]). In this study, progressive and massive necrosis of the tumor masses were observed, with a significant increase of the survival expectation and one case of complete remission lasting over a 18 month period. IL4 receptor overexpression has been then confirmed in many other brain tumors [102] making this receptor a good general target for this pathological tissues. Interestingly, IL4 receptor, as well as transferrin receptor, expression is enhanced (from 25% to 45% with respect to the control, [103]) upon radiation therapy, thus suggesting a possible application of both radio- and toxin-based therapies for the treatment of glioblastoma. Along the same framework of interactions between IL receptors, it has been described in glioblastoma cells the presence of a variant of IL13 receptor (IL13R2, [104]) that is not present on normal brain tissues. The fusion toxin between IL13 and a truncated form of pseudomonas exotoxin proved to be very efficient in killing glioblastoma cells in culture [105, 106] and in human xenografts on nude mice [107]. Furthermore, a mutated form of human IL13 has been engineered to bind the IL13R2 with very high affinity with respect to wt IL13R, thus allowing the creation of new fusion toxins [108] practically devoid of any aspecific toxicity. IL13-based toxin have been introduced into clinical trials [108] and demonstrated to be effective in inducing tumor necrosis. Finally, the urokinase-type plasminogen activator receptor represents a good candidate for targeted therapies [109, 110], this receptor being fundamental for the activation of invasive processes. A very promising and interesting immunotoxin has been created by the fusion of the IL13 molecule with the N-terminal fragment of human urokinase (ATF) and the toxic and translocation domains of diphtheria toxin (DTAT3, [111]), able to induce complete killing of small tumors in mouse xenograft models.

## 6. Differentiating and Proapoptotic Therapies

### 6.1. Retinoic Acid

All transretinoic acid (ATRA) is an important modulator of multiple biological processes [112]. It has been shown that ATRA induces morphological changes well-matched with differentiation, suppresses proliferation, and even causes apoptosis in some tumor cells, including glioblastoma cells [113–115]. In tumor cells, ATRA treatment may result in increases in p21, p27, and p53 protein levels and cell-cycle arrest at G1 phase, which also correlates with significant downregulation of cell surface Her2/neu oncoprotein expression [112, 115]. It is well documented that downregulation of cell surface Her2/neu expression reverses transformed phenotypes and leads to a reduction in proliferation of tumor cells. Treatment of tumors with ATRA has been shown to exhibit increased sensitivity to MHC class I-restricted killing by CTL and NK-cell-mediated lysis [116, 117]. ATRA has also been shown to be beneficial in leukemia, cervical cancer, thyroid cancer, breast cancer, squamous cell carcinoma, skin cancer, and head and neck cancer when administered alone or in combination with other therapies [118]. Moreover, ATRA may induce the expression of proteolytic and regulatory subunits of the immunoproteasome, increase the half-life of MHCclass I complexes, and enhance the sensitivity of tumor cells to both MHC class I-restricted peptide-specific and MHC nonrestricted lysis by CTL, NK, and NK T-cells [119–121]. ATRA also induces systemic modulation of antigen presentation by nonprofessional antigen presenting cells such as tumor cells. In addition, ATRA has been shown to modify the immunogenicity of tumor cells both in vitro and in vivo through differential regulation of MHC class I and intercellular adhesion molecule-1 (ICAM-1) [114, 117]. The upregulation of ICAM-1 may increase the sensitivity of glioblastoma to NK-cells. Studies suggest that tumor cells can be converted to efficient antigen presenting cells for direct antigen presentation and T-cell stimulation [120]. It has been has shown that IFN-*γ* is an important biomolecule for positive regulation of the MHC presentation machinery. The treatment of glioblastoma cells with IFN-*γ* induces apoptosis and the extent of cell death is enhanced by pretreatment with ATRA. It was also showed that a combination of ATRA and IFN-*γ* expressed higher levels of HLA class II and HLA-DM molecules in glioblastoma T98G and U87MG cells than IFN-c alone [121], suggesting that the combination of ATRA with INF-*γ* may overcome the defect in class II-mediated immune recognition of glioblastoma [121].

### 6.2. PPAR*γ* Agonists

The peroxisome proliferator-activated receptors (PPARs) are a subgroup of ligand-activated transcription factors. They belong to the nuclear receptor family. Other members of this family include steroid and thyroid hormone receptors, retinoid receptors, and vitamin D receptors [122]. The PPAR family comprises three closely related gene products, PPAR*α*, *β*/õ, and *γ*. All have a highly conserved structure [122]. PPAR*γ* activation plays a role in diverse physiological and pathophysiological events including stimulation of adipocyte differentiation, activation of insulin, regulation of lipid metabolism, inhibition of tumor cell proliferation, and diverse effects on inflammatory processes [122]. Endogenous PPAR*γ* ligands are polyunsaturated fatty acids and eicosanoids, such as 15-deoxy-delta 12,14-prostaglandin J2 and leukotriene B4. A number of synthetic PPAR*γ* ligands have been identified over the past 7 years, of which the most well known are the thiazolidinediones (TZDs) (pioglitazone, ciglitazone, rosiglitazone, etc.). TZDs are a class of antidiabetic agents that improve insulin sensitivity and reduce plasma glucose and blood pressure in patients with type2 diabetes mellitus [122]. It is well known that the response of tumor cells to antiproliferative treatments is strongly dependent on their differentiation degree. PPAR not only plays a crucial role in apoptosis but also in differentiation of a variety of cell types, including malignant cells (since induction of differentiation through PPAR activation has been observed in several malignant cells; [123–125]. Several studies have reported antiproliferative and/or differentiating activities of some lipophilic molecules on glioblastoma cells. Some of these activities in cell signaling are mediated by PPARs [126]. PPAR has been identified in transformed neural cells of human origin, and it has been demonstrated that PPAR agonists decrease cell proliferation, stimulate apoptosis and induce morphological changes and expression of markers typical of a more differentiated phenotype in glioblastoma and astrocytoma cell lines [127]. These findings arise from studies mainly performed on long-term cultured transformed cell lines and more recently also in glioblastoma primary cultures. It has been reported that PPAR*γ* natural and synthetic ligands may interfere with glioblastoma growth and malignancy and might be taken in account as novel antitumoral drugs. In fact, treatments with natural or synthetic ligands of PPAR*γ* decreases the expression of undifferentiating markers such as CD133, nestin, and fibronectin while increasing the expression of differentiation markers such as A2B5, GFAP, *β*-catenin, and N-cadherin. CLA and PPAR*γ* agonist suppress proliferation and induce apoptosis in primary cultures of glioblastoma cells [128]. Consistently with growth inhibition, both ligands downregulate cyclinD1and CDk4 protein levels, while inducing the transcription of the tumor suppressor gene PTEN. Besides being utilized for glioma histological characterization, astroglial marker GFAP is also recognized as an indicator of glioma differentiation, since its expression increases upon several anticancer drug treatments [128]. CLA and PPAR*γ* synthetic agonist induced a significant increase of GFAP protein levels as well as the acquirement of the astrocytic phenotype indicating that activated-PPAR*γ* induces differentiation of glioblastoma cells. Both CLA and PPAR*γ* agonist treatments led to a significant decrease of the VEGF isoforms and NOSII, thus indicating that even in glioblastoma, PPAR*γ* is able to inhibit the angiogenetic pathways [128]. The overall, the data in the literature point towards the possibility to use PPAR*γ* ligands in the treatment of malignant gliomas and their recurrence.

### 6.3. Autophagic Drugs

Proautophagic drugs are a promising class of compounds in counteracting tumor progression by favouring cell death [129–131]. A variety of chemical or physical treatments, including rapamycin (mTOR inhibitor) [132–134], arsenic trioxide [135], ceramide [136], temozolomide [137, 138], dopamine [139], endostatin [140], the histone deacetylase (HDAC) inhibitors butyrate and suberoylanilide hydroxamic acid [141], neodymium oxide [142], and resveratrol [143], have been reported to induce autophagy in vitro and in vivo in certain cancer cells. Rapamycin and its analogues (such as CCI-779, RAD001, and AP23573) inhibit mTOR, the kinase that normally suppresses both apoptosis and autophagy and that is active when nutrients are abundant. Rapamycin activates the autophagic process [129–131], and the inhibition of autophagy by small interfering RNA (siRNA), directed against the autophagy-related gene beclin 1, attenuates the cytotoxicity of rapamycin in rapamycin-sensitive tumor cells, indicating that autophagy is a primary mediator of rapamycin-mediated antitumor effects rather than a protective response [144]. Exogenous expression of an mTORmutant, interfering with its kinase activity, markedly enhances the incidence of rapamycin-induced autophagy [144]. Importantly, not only rapamycin-sensitive malignant glioma cells but also rapamycin-resistant malignant glioma cells with wild-type PTEN are sensitized to rapamycin by mTOR siRNA [144]. In addition, mTOR inhibitors sensitize tumor cells to DNA-damaging agents in vitro [134].

## 7. Future Directions in Glioma Therapy

The progress and depth of understanding of the biology and genetics of glioma, together with truly manipulable experimental models, now offer very real opportunities for the development of effective targeted therapy. These new approaches will, in the future, integrate the current experimental therapies. Despite significant gaps in our understanding, a wealth of information now exists about the clinical and biological behavior of the tumors, the genetic pathways involved in gliomagenesis, and the nature and role of signature alterations in these pathways. The challenge now is to integrate all of this knowledge in an interdisciplinary way to fully understand this disease and how its signature heterogeneity contributes to its intractability.
